# Immune Responses in Leishmaniasis: An Overview

**DOI:** 10.3390/tropicalmed7040054

**Published:** 2022-03-31

**Authors:** Ana Caroline Costa-da-Silva, Danielle de Oliveira Nascimento, Jesuino R. M. Ferreira, Kamila Guimarães-Pinto, Leonardo Freire-de-Lima, Alexandre Morrot, Debora Decote-Ricardo, Alessandra Almeida Filardy, Celio Geraldo Freire-de-Lima

**Affiliations:** 1 Instituto de Biofísica Carlos Chagas Filho, Universidade Federal do Rio de Janeiro, Rio de Janeiro 21944-970, Brazil; anaccsilva@gmail.com (A.C.C.-d.-S.); leolima@biof.ufrj.br (L.F.-d.-L.); 2 Instituto de Veterinária, Universidade Federal Rural do Rio de Janeiro, Seropédica 23890-000, Brazil; daniongabi@gmail.com (D.d.O.N.); decotericardo@ufrrj.br (D.D.-R.); 3 Instituto de Microbiologia Paulo de Góes, Universidade Federal do Rio de Janeiro, Rio de Janeiro 21944-970, Brazil; rafamachadoferreira283@gmail.com (J.R.M.F.); milakgp@gmail.com (K.G.-P.); filardy@micro.ufrj.br (A.A.F.); 4 Faculdade de Medicina, Universidade Federal do Rio de Janeiro, Rio de Janeiro 21941-900, Brazil; alexandre.morrot@medicina.ufrj.br; 5 Instituto Oswaldo Cruz, FIOCRUZ, Rio de Janeiro 21045-900, Brazil

**Keywords:** leishmaniasis, infection, immunology, immunoparasitology, immunomodulation

## Abstract

Leishmaniasis is a parasitic, widespread, and neglected disease that affects more than 90 countries in the world. More than 20 *Leishmania* species cause different forms of leishmaniasis that range in severity from cutaneous lesions to systemic infection. The diversity of leishmaniasis forms is due to the species of parasite, vector, environmental and social factors, genetic background, nutritional status, as well as immunocompetence of the host. Here, we discuss the role of the immune system, its molecules, and responses in the establishment, development, and outcome of Leishmaniasis, focusing on innate immune cells and *Leishmania major* interactions.

## 1. Leishmaniasis

Leishmaniasis is a neglected endemic tropical disease distributed in more than 90 countries throughout the New World (Latin America) and Old World (Africa, Asia, and Southern Europe), primarily found in Southeast Asia, East Africa, and Brazil. It has an estimated prevalence of 12 million cases worldwide, which is continuously growing, with 1.5–2 million new cases each year [1,2]. Leishmaniasis is an infectious disease caused by parasites of the genus *Leishmania*, of the Trypanosomatidae family. This disease manifests in four main forms: cutaneous leishmaniasis (CL), diffuse cutaneous leishmaniasis (DCL), mucocutaneous leishmaniasis (MCL), and visceral leishmaniasis (VL) or kala-azar. CL, the most common form, causes non-lethal skin lesions and is primarily caused by *L. major*, *L. tropica*, and *L. aethiopica* in the Old World, or by *L. mexicana*, *L. amazonensis*, *L. braziliensis*, *L. panamensis*, and *L. guyanensis* in the New World [3]. DCL, the anergic form of CL, is a rare condition in which lesions are full of parasites, and is characterized by multiple nodules, papules, or tubercles with diffuse skin infiltration and no ulceration. The main causative species of DCL are *L. mexicana* and *L. amazonensis* in the New World; and *L. aethiopica* in the Old World. VL, the most severe clinical form of leishmaniasis, is responsible for thousands of deaths each year and is caused by *L. donovani* and *L. infantum* in the Old World or *L. chagasi*; and by *L. infantum* in the New World. This form is characterized by prolonged fever, splenomegaly, hypergammaglobulinemia, and pancytopenia. Finally, MCL is mainly caused by *L. braziliensis* and eventually by *L. panamensis* or *L. guyanensis*. About 90% of MCL cases occur in Bolivia, Brazil, and Peru. This form of leishmaniasis is characterized by the dissemination of the infection from primary cutaneous lesions to the mucosal system via direct extension, bloodstream, or lymphatics [4,5,6].

The transmission of the protozoan to humans or other mammals occurs through the bite of the female sandfly belonging to the order Diptera, family Psychodidae, and subfamily Phlebotominae of the genera Phlebotomus (Old World) and Lutzomyia (New World). *Leishmania* has a relatively simple heteroxenic life cycle: an extracellular promastigote stage, which can be either procyclic, which multiplies and develops in the digestive tract of sandflies, or metacyclic infective, which migrates to the proboscis of the sandfly to be inoculated during the blood meal; as well as an intracellular stage in the form of a spherical, immobile amastigote, which is morphologically and biochemically distinct from promastigotes, and resides and multiplies in the phagolysosomes of phagocytes in their vertebrate hosts [7,8].

**Figure 1:**
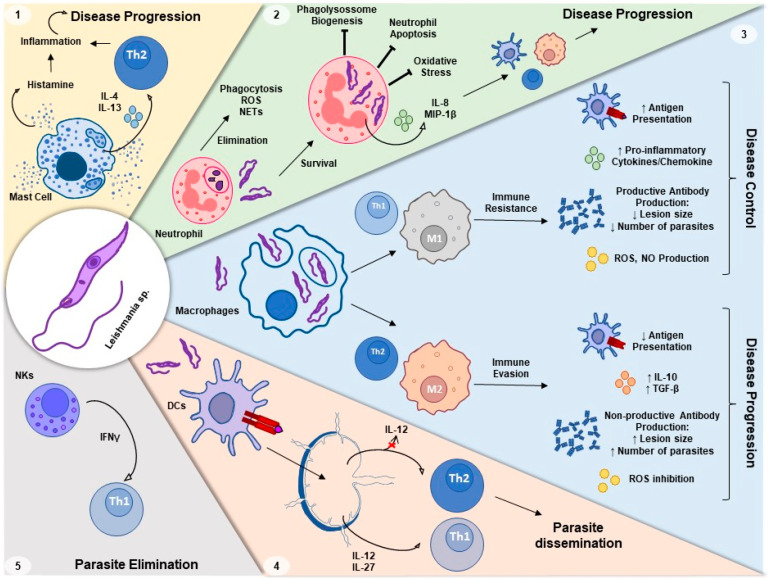
Impact of *Leishmania* infection on immune cells. *Leishmania* spp. interacts with multiple innate immune cells modulating their phenotype and function, as well as the adaptive immune responses. Mast cells collaborate in disease progression by secreting IL-4 and IL-13 fostering Th2 responses and parasite survival (panel 1). Neutrophils, macrophages, and DCs can either eliminate or promote parasite survival. Recruited neutrophils eliminate leishmanial parasites through phagocytosis, ROS, and NETs release. *Leishmania* can survive transiently within neutrophils by inhibiting phagolysosome biogenesis and oxidative stress, and by delaying neutrophil apoptosis. Infected neutrophils also secrete IL-8 and MIP1β, which attract additional neutrophils and other phagocytic cells, favoring *Leishmania* survival and pathology (panel 2). Macrophages can be differentiated in M1 or M2 during leishmaniasis. M1 macrophages produce proinflammatory cytokine and chemokines, NO and ROS, booster Th1 responses, and favor disease control. M2 macrophages increase the production of IL-10 and TGFβ, and support Th2 response and disease progression (panel 3). DCs regulate immune responses against *Leishmania* by migrating to the draining lymph nodes to present *Leishmania*-derived antigen to naïve T cells. DCs can induce the differentiation of Th1 by secreting IL-12 and IL-27 or Th2, by blocking IL-12 secretion (panel 4). NK cells have a protective role in leishmaniasis by secreting IFNγ to boost Th1 response (panel 5). IL; interleukin IFNγ; ROS; reactive oxidative species, NO; nitric oxide, NETs; neutrophil extracellular traps, MIP; macrophage; inflammatory protein, TNFα; tumor necrosis factor α, TGFβ; transforming growth factor-β, NK; natural killer.

Recent advances in imaging technologies, as well as studies combining genetic and immunological manipulations, have allowed a better understanding of the interactions between *Leishmania* and its mammalian host, especially the role of different cell types involved in the initiation and development of the immune (Figure 1) [7,9]. The divergent clinical manifestations observed in leishmaniasis are dependent not only on the genetic background, nutritional status, and immunocompetence of the host, but also largely on the species of parasite that initiates the infection, the vector, and environmental and social factors [10].

## 2. Th1 versus Th2 Response to *Leishmania* Infection

The balance between the type 1 and type 2 responses, along with regulatory mechanisms, is a determinant of the outcome of leishmaniasis. The *L. major* mouse infection model has been widely used to decipher some of the immune response mechanisms involved in susceptibility and resistance to a parasitic infection, even though it does not apply consistently to all forms of leishmaniasis.

The cellular immunity generated by a Th1/Tc1 response is considered an important mediator of resistance to *Leishmania*. Subcutaneous infection with *L. major* promastigotes promotes a Th1 skewed protective response in resistant murine strains, such as C57BL/6, C3H, and CBA, leading to the development of small lesions that heal spontaneously, control of parasite replication, and immunity against reinfection [11,12,13]. On the other hand, an improperly modulated and exacerbated type 1 response can result in severe tissue damage and clinical presentation of leishmaniasis. CL caused by *L. tropica* [14] and MCL caused by *L. braziliensis* and *L. amazonensis* [15] are characterized by increased amounts of proinflammatory cytokines, including IFNγ and TNFα.

With regard to susceptible murine strains, such as BALB/c, the course of *L. major* infection is characterized by the development of a Th2 response with persistent inflammatory lesions, uncontrolled replication of the parasite, and its systemic dissemination to lymph nodes and the spleen [11,12,13]. Interestingly, a previous study demonstrated that Balb/c mice lacking IL-4Rα signaling in dendritic cells (DCs) are highly susceptible to *L. major* infection, pointing to the role of early IL-4R signaling on DCs for protection against CL [16].

In addition to Th1 and Th2 responses, other mechanisms are described as important for disease control or progression, depending on the species of *Leishmania* and the animal model employed. Increased levels of IL-17 as well as IL-10 have been described in patients with CL and MCL [17,18,19], implying the role of Th17 cells and regulatory T cells (Tregs) in the pathogenesis of leishmaniasis. Nonetheless, the role of these cells has been thoroughly discussed by others [20,21,22], and is not the scope of this review.

## 3. Innate Immune Response to *Leishmania* Infection

The immune response is initiated at the site of pathogen entry. Upon inoculation of *Leishmania* in the dermis, promastigotes interact with serum components, activating the complement system, both classical and alternative pathways. The opsonization of metacyclic promastigote forms by complement is rapid and efficient, resulting in approximately 90% lysis of the inoculated parasites. However, the parasites developed mechanisms that allowed them to resist and bypass this lysis process [23,24,25,26]. First, promastigote metacyclogenesis gives rise to more complement-resistant forms. Furthermore, *Leishmania* expresses protein kinases that phosphorylate C3, C5, and C9 components, inhibiting complement activation [27,28]. Finally, two molecules on the surface of the parasite, lipophosphoglycan (LPG) and glycoprotein of 63 kDa (GP63), mediate their binding to inactivated C3b (iC3b), preventing complement-mediated lysis, and facilitating the internalization of *Leishmania* via complement receptors (CRs) [29].

Sandfly saliva also contributes to the infection outcome. It contains exosomes [30], gut microbes [31], and molecules that have diverse activities including vasodilation, coagulation inhibition, and immunomodulatory effects [32]. Furthermore, it has already been described that the promastigote forms of *Leishmania* induce the secretion of MCP-1, CXCL1, and CXCL2 which act by attracting both monocytes and neutrophils [33,34]. Indeed, sustained recruitment of phagocytic cells to the bite site plays an essential role in infection establishment.

Resident cells, such as macrophages, keratinocytes, mast cells, and Langerhans cells, called sentinel and present at the inoculation site, express a variety of pattern recognition receptors (PRRs), including Toll-like receptors (TLRs), which recognize pathogen-associated molecular patterns (PAMPs) and trigger the phagocytosis of microorganisms and opsonized particles. These cells also express several cytokine receptors and, together with other tissue cells, produce several chemokines, initiating innate and acquired immune response activation cascades [33,35].

Activated cells undergo several morphological and functional changes that lead to parasite phagocytosis and oxidative stress. After their initial encounter with neutrophils, *Leishmania* parasites are phagocytosed by both monocytes and macrophages, and DCs [36]. Phagocytosis involves three distinct events: binding to specific receptors, engulfment with the formation of the parasitophorous vacuole, and parasite death or degradation by the formation of reactive oxygen species/reactive nitrogen species (ROS/RNS) [37,38]. These specific events are discussed further in a later section.

### 3.1. Neutrophils–Leishmania Interaction

Neutrophils are the first cells to arrive at the site of *Leishmania* infection and their role can be either beneficial or detrimental, depending on the *Leishmania* species and host factors [39]. Interestingly, in the murine model of CL, recruitment of neutrophils depends on whether the mouse strain is susceptible (BALB/c) or resistant (C57BLl/6) to the infection. Initially, both strains show a similar inflammatory response. However, neutrophils become prominent and persist in susceptible hosts, whereas this recruitment is not sustained in resistant animals, returning to basal levels within three days [11,40,41].

Infiltrating neutrophils target and eliminate leishmanial parasites through different mechanisms, including the phagocytosis and production of an array of intracellular and extracellular microbicidal factors such as ROS and neutrophil extracellular traps (NETs) [24,42]. However, *Leishmania* can survive transiently within neutrophils through several protective mechanisms, including the inhibition of phagolysosome biogenesis [43], the prevention of oxidative stress [44], and the delay in neutrophil apoptosis [45]. Besides, infected neutrophils also induce the production of a variety of chemokines and cytokines, such as interleukin (IL)-8 and MIP1β, which attract additional neutrophils and other phagocytic cells, favoring *Leishmania* survival and pathology [46,47,48].

In vitro studies suggest that *Leishmania* uses neutrophils as intermediary cells for the infection of its definitive host cell, in a model called “Trojan Horse” [47,49]. In this model, macrophages are able to phagocytose parasitized neutrophils, and the parasites survive and multiply within the macrophage vacuoles, even managing to multiply within macrophages. Phagocytosis of dead neutrophils can be both pro- and anti-inflammatory. In susceptible mice, such as BALB/c, phagocytic removal of apoptotic neutrophils (efferocytosis) exacerbates the growth of the parasite inside macrophages through the production of TGFβ and PGE2 [50,51]. Furthermore, the depletion of neutrophils inhibits the production of IL-4 and reduces the parasite’s ability to engage in productive cycles of infection and parasite burden, providing a better response to infection [40,49]. In contrast, the phagocytic removal of apoptotic neutrophils by macrophages from resistant C57BL/6 mice induces the death of the parasite mediated by the enhanced production of neutrophilic elastase and TNFα [52]. The transient depletion of these neutrophils at the time of infection, in turn, increases the growth of the parasite and the size of the lesion in resistant mice, even if, in this case, these mice eventually heal [40,50]. The definitive role of neutrophils in *Leishmania* infection outcome has yet to be defined with more comprehensive models employing different parasite species and genetic backgrounds.

### 3.2. Macrophage–Leishmania Interaction

The interaction between macrophages and *Leishmania* plays a fundamental role in the pathogenesis of the infection. In the experimental leishmaniasis models, macrophages are crucial cells not only for the survival, replication, and differentiation of the parasites, but also for their elimination [53]. Macrophages correspond to the main reservoir of *Leishmania* in vivo. Although it has already been demonstrated that these cells are not capable of producing IL-12 after infection by *L. major* [54], macrophages are capable of producing pro-inflammatory cytokines at the site of infection and killing and eliminating parasites through the production of nitric oxide (NO) [55]. They also induce the recruitment of pro-inflammatory cells, in addition to presenting *Leishmania* antigens to the T cells, along with DCs for primed T cells.

### 3.3. Dendritic Cells–Leishmania Interaction

DCs play an essential role in the initiation and regulation of effective immune responses against *Leishmania*. These cells can uptake and process antigens; mature and upregulate MHCII and co-stimulatory molecules; migrate to lymph nodes; and activate T cells to differentiate into effector Th1 cells through the production of IL-12 and IL-27 [56,57,58]. Distinct DCs subsets have been identified in the skin and several studies have shown their different functions in T cell activation and disease outcome.

Langerhans cells (LCs) were initially considered essential for the control of cutaneous leishmaniasis [59]. However, later studies demonstrated that these cells were not required for the generation of protective immunity against *Leishmania* parasites [60]. In fact, it was demonstrated that these cells can have a regulatory function, once the depletion of LCs dampens the production of IL-10 and the numbers of Tregs and favors the production of IFNγ and the decrease in parasite load [61]. Instead of LCs, the participation of dermal DCs (CD11c^+^ CD8α^−^ Langherin^−^) and monocyte-derived DCs proved to be essential for the transport of *Leishmania* antigens from the infection site to the draining lymph node and in the induction of a specific T cell response [62,63]. Interestingly, IL-4, a type 2 cytokine, is required for the optimal induction of Th1 responses by DCs as it inhibits IL-10 production [64,65].

### 3.4. Leishmania Interaction with Other Innate Immune Cells

Dendritic cells are not the only cell type needed and involved in developing an effective T cell response. Some studies demonstrate that natural killer (NK) cells have a protective role in leishmaniasis, being the primary source of IFNγ for the development of a Th1-type response [66,67]. Indeed, reduced numbers of NK cells and activation markers, such as IFNγ and TNFα, were observed in patients with diffuse cutaneous leishmaniasis compared to localized CL [66]. Recently, a contribution of NK cells to the immunopathology of CL was also demonstrated mainly through the release of granzyme B. It was suggested that NK cells can contribute to cytotoxicity activity in CL patients [68].

Mast cells are also present at the site of parasite inoculation and seem to be important to the outcome of leishmaniasis. These cells produce a variety of mediators and cytokines, participating in several stages of the innate and adaptive immune response. However, the precise role of these cells in *Leishmania* infection is not well understood [69]. Infection of mast cell-deficient C57BLl/6 mice with *L. major* demonstrated a protective role for these cells, since their absence led to the development of larger skin lesions containing greater amounts of parasites and also an increase in the spread of these parasites to the spleen [70,71]. On the other hand, other studies suggest that mast cells play a deleterious role in *Leishmania* infection, through the production of IL-4 and IL-13 and the establishment of Th2 response [72,73]. Furthermore, mast cells’ degranulation before *L. major* infection showed their ability to regulate not only inflammation through histamine release, but also to promote Th2 response [74].

Keratinocytes have already been described as having an important role in the initiation of infection, acting not only as a physical barrier, but also secreting molecules that can shape the immune responses to the pathogen. For example, it has already been reported that keratinocytes are capable of secreting immunomodulatory mediators such as IL-12, IL-1β, osteopontin, IL-4, and IL-6 [75].

## 4. *Leishmania* and Host Immune Receptors’ Interaction and Intracellular Processing of *Leishmania*

The first crucial event for *Leishmania* infection involves the initial contact and stable interaction between the promastigote forms of the parasite and host cells. Recognition and phagocytose of promastigotes can be mediated by several molecules including CR1, CR3 (Mac-1), fibronectin receptor (FnR) and C-type lectin receptors (CLRs) expressed on the surface of macrophages [74]. However, it has been demonstrated that the individual binding of these receptors does not lead to macrophage activation, suggesting that multiple receptors may be important for the initiation of the appropriate immune response [75].

The interaction of *Leishmania* with macrophages primarily occurs through CR1 and CR3 receptors that recognize iC3b-opsonized parasites [76,77]. Inactivation of C3b molecules is a defense mechanism developed by *Leishmania* that allows its silent entry into macrophages with no induction of oxidative stress [77], and reduced production of IL-12 [78]. Mechanisms of C3 molecule inactivation have been attributed to the protease gp63 [79] and uptake of factor H, a regulatory molecule of the complement [26].

FnR cooperates with CR3 for binding and phagocytosis of promastigotes. Previous studies demonstrated that this receptor increases *Leishmania* binding to phagocytes [80,81,82], but, interestingly, FnR also decreases *Leishmania* intracellular survival [82].

Some conflicting data have been observed regarding the participation of CLRs, such as Dectin-1, mannose, and Mincle receptors, in the internalization of promastigote forms of *Leishmania* by macrophages and dendritic cells [83].

The precise role of *Leishmania* binding to MR, whether pro- or anti-inflammatory, is still unclear. *L. donovani* promastigotes’ interaction with monocyte-derived macrophages was partially inhibited by the addition of specific MR ligands and monoclonal antibodies against the receptor [84]. Stimulation of MR promoted an effective immune response and clearance of *L. infantum* infection [83]. However, the use of MR-deficient animals showed that the phagocytosis of metacyclic promastigotes of both *L. major* and *L. donovani* occurred efficiently and similarly to that in wild-type animals [80].

Dectin-1 also promoted oxidative stress production and clearance of *Leishmania* in a VL murine model [83]. An increased expression of Dectin-1 was also observed after macrophages’ infection with *L. amazonensis* in vitro [84] and, more recently, the expansion of Dectin-1 + DCs was also observed in experimental leishmaniasis as well as in patients suffering from CL [81].

Importantly, SIGNR3 expression in macrophages favored *L. infantum* resilience through decreased IL-1β expression, whereas Mincle was shown to promote the infection of *L. major*, by dampening dendritic cell priming through the activation of the inhibitory ITAM pathway [82].

Despite the large amount of information available about the phagocytosis process of the promastigote forms, few studies have been conducted on the phagocytic processes of the amastigote forms, which are responsible for the maintenance and dissemination of the infection in the vertebrate host. Promastigote forms encounter macrophages only in the early stages of infection, whereas amastigotes are continuously released from infected cells and are internalized by other uninfected cells, giving rise to a progressive infection. In addition, opsonized amastigotes are phagocytosed via FcγR, a process that results in IL-10 secretion, facilitating the survival and replication of the parasite. [85,86,87,88] In fact, BALB/c JHD (deficient in circulating antibodies) or FcγR-/- animals have smaller skin lesions after infection by *L. amazonensis* and *L. pifanoi* [89].

It is also worth mentioning that DCs preferentially internalize the amastigotes of the parasite via FcγR [90,91] and, therefore, the infection of these cells occurs in the late stages of infection, when *L. major* amastigotes are released into the tissue. This scenario provides plausible explanations for the delay that occurs between parasite inoculation and the development of a cellular immune response that is observed in cutaneous leishmaniasis.

In addition to FcRs, other receptors participate in the phagocytosis of amastigotes, such as CRs, FnR, and heparin-binding protein, and it was also seen that binding to the MR, in this case, did not affect the entry of amastigotes [92].

Although surface receptors initially determine the phagocytosis of *Leishmania* and its route within host cells, the internalized parasites continue to apply strategies to promote their survival. Besides being crucial for the initiation of phagocytosis and subsequent intracellular survival of the parasite, *L. major* surface molecules such as LPG, gp63, and proteophosphoglycans can also act as ligands for different TLRs [93].

TLRs play an important role in the recognition of pathogens and activation of immune cells. Importantly, TLR2, TLR4, and TLR9 have been described as important mediators of immune cells and *Leishmania* interaction [94]. Mice lacking TLR signaling are more susceptible to *L. major* infection, presenting a decreased ability to induce protective immunity and increased parasite burden [95,96]. In contrast, the absence of TLR signaling in mice infected with *L. amazonensis* resulted in the improvement of immune responses with a higher production of IL-12 and enhanced resistance to infection [97]. More recently, decreased levels of inflammatory molecules and reduced parasitic load were observed after the neutralization of TLR2 and TLR4 in CL patient monocytes [92]. TLR9-dependent activation of macrophages [98], dendritic cells [99], and NK cells [100] is also important for the resolution of *Leishmania* infection.

After the internalization of the promastigote forms into phagosomes, lysosomes fuse for the complete formation of the parasitophorous vacuole. Walker et al. demonstrated that LPG impairs vacuole acidification by inhibiting the functional assembly of the NADH oxidase complex and preventing vacuolar proton recruitment ATPase on the parasitophorous membrane. This process not only allows the transformation of the parasite into amastigotes [101], but also inhibits the activation of lysosomal proteases necessary for antigen processing and the initiation of the immune response [43].

Unlike promastigotes, amastigotes are able to survive inside the phagolysosome because, among other things, they have proton pumps in their plasma membrane that capture metabolites and also metabolite transporters whose functions are properly exercised at acidic pH [102]. The zinc-metalloprotease GP63, or leishmaniolysin, the most abundant protein in amastigotes, is an endoproteinase whose proteolytic activity at an acidic pH is relevant for the survival of amastigotes in phagolysosomes, probably through the inactivation of lysosomal macrophage proteins [103,104].

In general, binding to specific receptors culminates in the activation of different pathways and functions in macrophages, and each interaction occurs because of factors expressed by the parasite itself. These virulence factors are regulated during the parasite life cycle and guarantee its ability to select the routes of immune system invasion and evasion [92].

## 5. Changes in Macrophages after *Leishmania* Infection

Changes in macrophages caused by *Leishmania* infection have been the subject of several studies to understand how the promastigote forms, after being internalized, are able to differentiate into amastigotes and multiply within a hostile environment such as the parasitophorous vacuole. Studies have shown that macrophages infected with *Leishmania* have a deficient response to IFNγ, characterizing them as deactivated macrophages [105]. Filardy et al. showed that the infection with *L. major* rapidly triggers a cellular stress response in residents, but not inflammatory peritoneal macrophages. This response induces proinflammatory signals, such as the secretion of the cytokines/chemokines TNF-α, IL-6, TIMP-1, IL-1RA, GCSF, TREM, KC, MIP-1α, MIP-1β, MCP-1, and MIP-2, but is also involved in parasite survival and replication in host macrophages [38]. Furthermore, the critical involvement of signaling through MyD88 and TLR in protecting against *L. major* infection is well established [106].

The first study to characterize which genes were modulated by *Leishmania* infection in macrophages from BALB/c mice revealed that 37% of the analyzed genes, totaling 588 genes, including the CD40 gene, were downregulated in infected macrophages with *L. donovani*, when compared to non-infected macrophages not infected with *L. donovani*. In contrast, only eight of the messenger RNAs (mRNA) studied had their expression increased, some of them linked to the recruitment of more macrophages to the site of infection, including MIP-1α and MIP-1β genes [107]. Dillon et al. also aimed to identify global changes in gene expression using murine macrophages from C57BL/6 mice and *L. major* at 4, 24, 48, and 72 h post-infection [108]. They demonstrated that genes related to both pro- and anti-inflammatory immune responses and glycolysis were substantially upregulated, and genes related to lipid metabolism, biogenesis, and Fc gamma receptor-mediated phagocytosis were downregulated in the murine macrophages. Human monocytes infected with *L. major* also disclosed an upregulation of pro-inflammatory cytokine and cytokines receptors including IL1A, IL1RN, IL6, and IL6R [109].

Another study comparing *L. major* and *L. donovani* infection in human macrophages revealed that the profile of modulated genes differs between species of the same genus, probably resulting in the different clinical manifestations observed. In this study, negative modulation of a group of genes induced by IFNγ was observed in macrophages infected with *L. major*. This study, however, showed that similar amounts of mRNA were both up- and down-regulated by both *Leishmania* species [110]. Shadab et al. described that murine peritoneal macrophages infected with virulent *L. donovani* strains showed suppression of many important cellular processes, including protein synthesis, in comparison to non-virulent variants. Genes encoding virulence factors and those important for parasite survival were significantly upregulated in the intracellular virulent amastigotes. In contrast, genes involved in the immune stimulations and negative regulation of the cell cycle and transcriptional regulation were also all upregulated in the non-virulent strains [111].

Rodriguez et al. demonstrated in a model of macrophage infection of BALB/c animals with *L. chagasi* the negative modulation of several pro-inflammatory genes, such as phagocytic receptors linked to classical macrophage activation—FcγRI and FcγRIIb. Furthermore, positive modulation of several genes associated with alternative activation of macrophages or Th2 response was observed, such as MR, CCR3, MIP-2, and TGF-βRII, among others [105].

Transcriptional analysis of macrophages from BALB/c mice infected with *L. amazonensis* amastigotes was also performed and the authors observed the induction of genes related to alternative macrophage activation, such as the increased expression of the arginase 2 gene, as well as IL-1Ra and the reduced expression of the CD14 gene [112]. Corroborating this work, the results of an experimental system with dual RNA sequencing of enucleated fibroblasts (cytoplasts) and intracellular *L. amazonensis*, which was performed recently to obtain further insights into parasites’ control over the host cell, suggested that a parasite-mediated control of the host cell transcripts’ half-life was beneficial to the parasite’s intracellular multiplication and evasion of the host immune response [113].

The gene expression profile of macrophages from C57BLl/6 and CBA mice was analyzed before and after infection by *L. amazonensis* and an increase in genes related to infection control in C57BLl/6 mice was reported, such as genes linked to apoptosis and phagocytosis, but not in CBA mice, where there was an increase in genes involved in lipid metabolism and, therefore, in the modulation of the parasitophorous vacuole [114].

Infection of peritoneal macrophages from C57BLl/6 mice with *L. amazonensis* and *L. major* demonstrated a non-generalized suppression of the lipopolysaccharide (LPS)-induced inflammatory response. It was observed that there was a reduction in the protein production of the cytokines IL-17, IL-12, IL-6, IL-13, and IL-3 induced by LPS, but an increase in TNF, IL-1α, MIP-1α, and MCP-1 [115]. In addition, the uptake of infected apoptotic cells induces the production of renders macrophages to produce transforming growth factor-β (TGF-β), creating an anti-inflammatory environment that promotes promoting *L. major* growth in macrophages [47]. Similar results were also presented with *L. amazonensis* by Afonso et al. [51].

More specifically, to survive within the hostile environment of macrophages, *Leishmania* has developed strategies to subvert the antimicrobial mechanisms mounted by these cells. These include the impairment of antigen presentation and cytokine secretion, sequestration of macrophage metabolic pathways, inhibition of NO, and induction of immunosuppressive molecules such as IL-10 and TGF-β; both cytokines involved in the deactivation of macrophage functions [116]. Furthermore, the induction of immunomodulatory molecules, such as CD200, by *Leishmania* also inhibits macrophage activation. In this same work, it was also shown that CD200-dependent inhibition of inducible nitric oxide synthase (iNOS) was responsible for the increased virulence of *L. amazonensis* [93,117].

In the context of the modulation of macrophage activation by *Leishmania*, studies in murine leishmaniasis models have shown that antibody production is associated with an unproductive immune response. Antibodies promote increased lesion size and parasite numbers during ineffective immunity towards *L. major*, *L. mexicana*, and *L. amazonensis* [88,118,119]. It was observed, in the study by MILES et al., that the presence of opsonized *Leishmania* seems to induce the production of IL-10, allowing the progression of the disease [88]. Furthermore, another study also demonstrated that the phagocytosis of apoptotic neutrophils by macrophages from C57BLl/6 mice leads to the induction of an M2b profile permissible for the *L. major* growth [120]. These data are reinforced by the observation of the relationship between the clinical phenotype of leishmaniasis and the magnitude of macrophage infection. Macrophages from individuals with chronic dermal leishmaniasis or recurrent disease were more permissive to *Leishmania* parasites than those from asymptomatically infected individuals [121,122]. These studies suggest that there may be an association between the response to *Leishmania* and the cell stage evaluated, with differentiated macrophages being more permissive to infection in vitro than monocytes [123]. In this sense, depending on the phenotype of monocytes/macrophages recruited to the site of infection and the infecting parasite species, these cells may contribute in different ways to host protection or disease immunopathology [124].

## 6. *Leishmania* and Oxidative Stress

The induction of cellular stress in *Leishmania*-infected macrophages is still a controversial topic. However, an increasing number of works indicate that *Leishmania* infection induces ROS production in macrophages [125,126] and several studies demonstrate the susceptibility of *Leishmania* to exogenous ROS and NO [4,127].

ROS are highly reactive oxygen-containing molecules, which can be free radicals (superoxide anion -O_2_-; hydroxyl radicals -OH; peroxynitrite -ONOO-, among others) or neutral molecules (hydrogen peroxide -H_2_O_2_; hypochlorous acid -HOCl; ozone -O_3_; singlet oxygen-^1^O_2_), among others [128]. ROS are known to activate various signal transduction pathways including the mitogen-activated protein kinases (MAPK), extracellular signal-regulated protein kinases 1 and 2 (ERK1/2), NF-κB, Nrf-2/Keap-1/ARE, and the PI3K/Akt pathway [129]. Several enzymes produce superoxide and other ROS, including the mitochondrial electron transport chain, NOS, cytochrome P450 oxidase, and xanthine oxidase. However, in all these systems, superoxide production occurs as a byproduct of other reactions. On the contrary, NADPH oxidase is the only enzyme whose primary function is the generation of superoxide/ROS and the main ROS-generating mechanism in macrophages [128].

Once produced, ROS can interact with a wide variety of biological molecules through electron donation, promoting cell signaling and regulation of numerous physiological processes including the regulation of immune cell functions and antitumor responses [130]. Several works described cell death because of NOX activation. Increased production of ROS is observed in cells that undergo apoptosis [131] and the use of exogenous ROS induces apoptosis in several cell types [132,133].

Upon recognition of *Leishmania*, properly activated macrophages become effector cells that can destroy phagocytosed pathogens through a variety of cellular processes including the production of lysosomal degradation enzymes, generation of oxidative stress, and production of NO [5,93]. Although *Leishmania* parasites are sensitive to ROS, the respiratory burst that occurs in non-activated macrophages following infection is insufficient to kill the parasites [134], which could be due to the parasites inhibiting ROS generation in phagolysosomes [135], even though both human and mouse monocytes produce high levels of ROS and can mediate ROS-dependent killing of *Leishmania* without prior activation [136,137]. Assreuy et al. demonstrated that the killing of *L. major* by IFNγ-activated murine macrophages in vitro was dependent on the production of NO, but not on the production of superoxide or peroxynitrite [138]. In humans, monocytes from patients with CL produced higher amounts of ROS after in vitro infection with *L. (V.) braziliensis* than healthy subjects. Moreover, the inhibition of ROS production in *Leishmania*-infected monocytes increased the presence of viable parasites, indicating their important role in parasite killing [139]. Gantt et al. demonstrated that both murine and human macrophages produce O_2_ and NO during phagocytosis of opsonized *L. chagasi* promastigotes and that both contribute to the intracellular death of the pathogen [125]. However, other studies demonstrate the opposite. Filardy et al. reported that *L. major* infection triggered a rapid cellular stress response, with increased production of ROS and proinflammatory signals, but is also involved in parasite survival and replication in host macrophages [38]. In addition, Blos et al. observed that Nox2-deficient animals infected with *L. major* controlled the infection in the initial moments, but they did not adequately control the replication of the parasite in the spleen, showing its importance in vivo at late moments [140].

The induction of suppressors of cytokine signaling (SOCS) proteins and thus apoptosis inhibition in *L. donovani*-infected macrophages was not affected by H_2_O_2_ treatment, which suggests the establishment of a replicative niche within the host [141]. Other studies also demonstrated the importance of ROS in the resistance to infection by *L. guyanensis* and *L. amazonensis* [142]. A possible role of superoxide and ONOO in the defense against *L. amazonensis* in vivo was also proposed [53,143,144].

## 7. Concluding Remarks

The importance of parasite–innate immune cell interactions became evident in both in vitro and in vivo models of *Leishmania* infection. We discussed the literature data on innate immune cells activity such as neutrophils, macrophages, and DCs, and the importance of their receptors and molecules in controlling *Leishmania* infection. In addition, we described different mechanisms that favor *Leishmania* survival and proliferation. Changes in signaling pathways and cell phenotype, as well as the increased production of a series of pro- and anti-inflammatory mediators, may contribute in a systematic way to the negative modulation of the immune response in the initial moments of infection. These mechanisms emerged because of a long parasite–host co-evolutionary process.

Additionally, some contradictory data found in the literature may reflect several variables including different infection models and the species of *Leishmania* used. Thus, the intimate connection between infection and the tissue repair response opens unexplored lines of research. Further investigation of innate immune cells and *Leishmania* can lead to a better understanding of the infectious process and to better vaccines for leishmaniasis.

## Data Availability

Not applicable.
